# Bacteriuria in Pregnancy in a Danish Contemporary Cohort of Women

**DOI:** 10.1155/2020/8398537

**Published:** 2020-01-08

**Authors:** Vinnie H. Greve, Thomas Greve, Rikke B. Helmig

**Affiliations:** ^1^Department of Obstetrics and Gynecology, Aarhus University Hospital, Skejby, Aarhus, Denmark; ^2^Department of Clinical Microbiology, Aarhus University Hospital, Skejby, Aarhus, Denmark

## Abstract

**Introduction:**

The purpose of this study is to describe bacteriuria with regard to the uropathogens found in relation to the frequency of urine culture tests in a contemporary cohort of pregnant Danish women.

**Methods:**

A historical cohort study of 24,817 pregnant women registered in the Danish Fetal Medicine Database at Aarhus University Hospital, from 2010 to 2014. Social security numbers were linked to the microbiological database with the registration of 17,233 urine cultures in 8,807 women. Bacteriuria was defined as 1 × 10^5^ CFU/ml, with a maximum of two urinary pathogens. *Streptococcus agalactiae* (GBS) was included with 1 × 10^4^ CFU/ml. Data are presented as numbers and proportions in percent. Logistic regression on predictors are presented as crude and adjusted odds ratios (OR_c_/OR_a_) with 95% confidence intervals (CIs).

**Results:**

42% had a urine sample culture test at the hospital—the majority only once during pregnancy. 96% of all urine culture tests were negative. The bacteriuria incidence was 5.6%. The most frequent uropathogenic bacteria isolated were *Escherichia coli* (49%), GBS (29%), and Enterococci (10%). We identified subgroups of women with increased likelihood of bacteriuria during pregnancy: age < 25 years, OR_a_ 1.60 (CI 1.26 to 2.02, *p* < 0.001); age > 34 years, OR_a_ 1.28 (CI 1.01 to 1.61, *p* = 0.040); Afro-Caribbean origin, OR_a_ 1.872 (CI 1.13 to 3.07, *p* = 0.014); Asian origin, OR_a_ 2.07 (CI 1.29 to 3.32, *p* = 0.002); and mixed ethnicity OR_a_ 2.34 (CI 1.23 to 4.46, *p* = 0.010). Women delivering preterm were more likely to have an episode of bacteriuria during pregnancy OR 2.05 (CI 1.36 to 3.09, *p* = 0.001).

**Conclusions:**

96% of urine culture tests in pregnant women are negative. Optimized urine sampling may change this*. Escherichia coli* and GBS are predominant uropathogens. Younger and elder women, certain ethnical groups, and women delivering preterm seem more likely to have bacteriuria during pregnancy.

## 1. Introduction

A urinary tract infection is a very common condition during pregnancy. It manifests itself as asymptomatic bacteriuria, cystitis, or pyelonephritis. Asymptomatic bacteriuria is defined as significant bacteriuria with a minimum of 10^5^ colony-forming units (CFU) of the same bacteria per milliliter of urine in two consecutive voided cultures in a person without any symptoms [1]. Cystitis is a clinical diagnosis with bacteriuria, with 10^3^ to 10^5^ CFU, depending on the bacteria found, together with symptoms such as dysuria, urgency, frequency, hematuria, suprapubic discomfort, or uterine contractions [1]. Pyelonephritis is diagnosed when symptoms such as fever, chills, flank pain, nausea, vomiting, and costovertebral angle tenderness are present together with bacteriuria and pyuria [1].

If untreated, asymptomatic bacteriuria and cystitis can progress to pyelonephritis [1–4]. Pyelonephritis is a recognized risk factor for adverse pregnancy outcome [5], but whether asymptomatic bacteriuria is a risk factor for preterm birth and low birth weight is still controversial and the literature is not conclusive on this matter [6–8].

The literature reports a prevalence of 2–10% of asymptomatic bacteriuria, 1–2% of cystitis, and ≤ 1% of pyelonephritis in pregnant women [1, 2, 7, 9–12] The prevalence of bacteriuria and pathogenic organisms causing bacteriuria are found to be similar in both pregnant and nonpregnant women. The predominant pathogen causing urinary tract infections in 70-80% of cases is *Escherichia coli* [1, 2, 13]. GBS is found in up to 10% of cases of bacteriuria [1, 11]. Reported risk factors of bacteriuria during pregnancy are low socioeconomic status, diabetes, multiparity, age, and history of urinary tract infections and functional urinary tract abnormalities [1, 2, 4, 10, 14].

The literature on bacteriuria during pregnancy is sparse, and there is no recent literature describing bacteriuria in a Danish pregnant population.

The current Danish antenatal guidelines are based on international recommendations and includes screening for asymptomatic bacteriuria to all pregnant women at their first and second visit to their general practitioner in gestational weeks 6–10 and 25.

Screening for bacteriuria can be performed either with urine dipsticks or urine microscopy, and if either is positive, regular urine cultures are recommended. There is no actual screening for *Streptococcus agalactiae* (GBS) in urine in Denmark. It is however recognized to be synonymous with heavy vaginal colonization, which constitutes a risk factor for neonatal infection during birth [15].

The purpose of this study was to describe the incidences of bacteriuria in a contemporary cohort of pregnant Danish women, to identify potential risk groups for bacteriuria, to determine the frequency of performing urine cultures during pregnancy, and lastly, to describe the uropathogens found in positive urine cultures.

## 2. Materials and Methods

This was a historical cohort study of pregnant Danish women registered for first trimester ultrasounds/nuchal scans at the Department of Obstetrics and Gynecology, at Aarhus University Hospital, Skejby, between January 2010 and December 2014. The department is a tertiary hospital in the second largest city in Denmark with 4,500–5,000 deliveries per year equaling one-third of all deliveries in the catchment area with approximately 14,500 deliveries per year. The pregnant population in Aarhus is in many aspects a low-risk group of pregnant women that are relatively young, and many are pregnant for the first time. Because it is also a tertiary hospital, it receives high-risk patients from the surrounding catchment area. In Denmark, we have approximately 62,000 deliveries every year.

The prenatal care is offered for free for every pregnant woman in Denmark. The standard prenatal care include consultation at the general practitioner in gestational weeks 8-10 and 25 with screening for asymptomatic bacteriuria. Nuchal scan and risk assessment are offered in gestational week 12 and ultrasound examination for malformations in week 20. The pregnant woman is offered 5 consultations with a midwife before term. If passing term, extra consultations including midwife, CTG, and ultrasound in week 41 + 3. Induction of labor is offered in week 41 + 5.

We used a unique social security number to link two different databases. The Danish Fetal Medicine Database (DFMD) holds information on all pregnant women receiving antenatal ultrasound examinations provided in weeks 12 and 20 as part of the public antenatal care in Denmark. More than 94% of pregnant women attend these ultrasound examinations in Denmark, and this pattern has been constant for years [16]. From the DFMD, we identified a total of 24,817 singleton pregnancies during this period. From the individual social security number, we collected information from DFMD describing the basic characteristics of the women such as age, ethnicity, and parity. Furthermore, we collected information about individual risk factors such as smoking habits and BMI together with information in relation to pregnancy outcome such as gestational age.

Data regarding the gestational age at birth in 100 pregnancies were missing, and these were therefore excluded from the cohort. We decided that only one pregnancy could be included from every woman in the five-year period. As a result, 3,783 pregnancies were excluded and a total of 20,934 pregnant women remained in the cohort. Social security numbers were linked to the Microbiological Database at the Department of Clinical Microbiology at Aarhus University Hospital (MADS), and for each woman, we extracted information on all quantitative urine cultures analyzed from conception until delivery. A total of 18,761 urine cultures were identified. We excluded 1,528 urine samples with less than 14 days between the dates of sampling, which would likely represent the same bacteriuria episode.

We defined urine culture to be positive when the colony count was at least 1 × 10^5^ CFU per ml of a single microorganism or when two different colony types were present, but one had a concentration of a least 1 × 10^5^colony-forming units per ml. When more than two species were present and the third in a considerable amount, the urine was judged to be contaminated. For GBS, we set a lower count of 1 × 10^4^ as significant growth as this bacterium is generally reported positive at lower counts. No growth, non-significant growth, or contamination was registered as culture negative in this study.

Species identification was performed using either chromogenic culture plates or MALDI-TOF-MS processing (Micro-flex™; Bruker Daltonics, Bremen, Germany). *E*. *coli* with a typical morphological presentation was identified using the chromogenic culture plate. All other species' identification was based on the phenotypical presentation and on a MALDI-TOF-MS score of more than 2.0 in accordance with the manufacturer's instructions.

### 2.1. Statistical Analysis

Logistic regression was used to examine the association between bacteriuria and no bacteriuria during pregnancy.

Based on clinically relevant data and review of the literature, we selected the predictors of bacteriuria from the DFMD database.

Univariable logistic regression was used on each stratum (maternal age, currently smoking, BMI, ethnicity, and parity). Statistically significant predictors were included in the multivariable analysis.

In each stratum, one subgroup was defined as the reference for comparison: maternal age of 25-34 years, non-smoking, BMI 20-24, Caucasian ethnicity, and nulliparous parity.

Data are presented as numbers and proportions in percentage. Predictors are presented as crude odds ratios (OR_c_) and for the multivariable analysis adjusted odds ratios (OR_a_) with 95% confidence intervals (CIs).

Stata/SE 15.1 for windows (StataCorpLP, Texas, USA) was used for statistical analysis.

### 2.2. Data Availability Statement

The study was approved by the Danish Data Protection Agency (j. number 1-16-02-288-12), the Danish Health Authority (j. number 3-3013-1701/1), and the Danish Fetal Medicine Database (j. number 2012-58-0023) in order to retrieve and analyze data.

The registered data used to support the findings of this study are restricted by the Danish patient safety authority in order to protect personal data. Anonymized data are available from the corresponding author upon request.

## 3. Results

**Figure 1:**
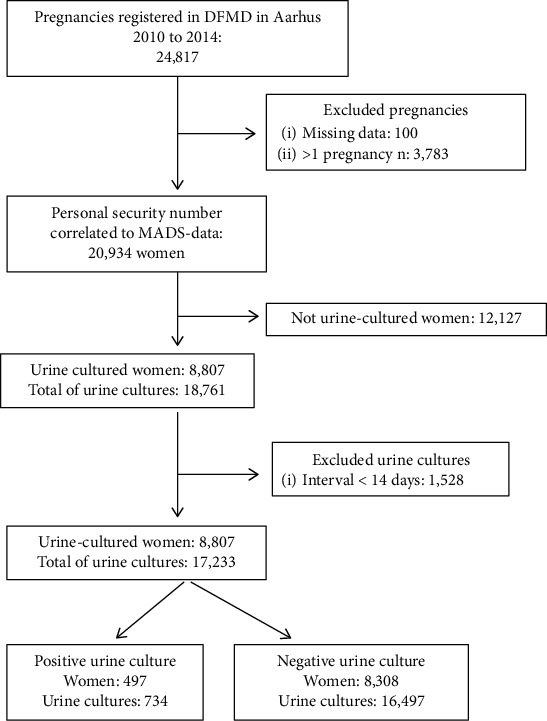
Included and excluded women and urine cultures.

Of the 20,934 women included, an overall 42% had a urine culture performed at least once during pregnancy, with a total of 18,761 urine cultures performed in the cohort (Figure 1).

**Figure 2:**
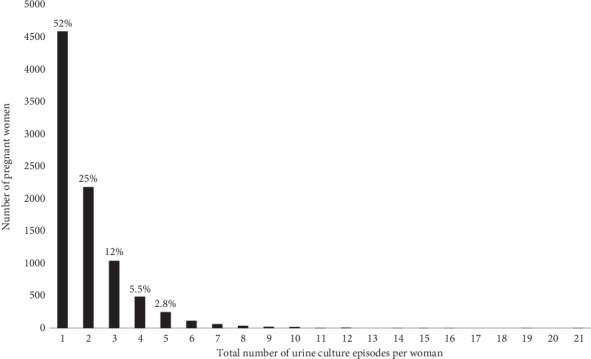
The number of pregnant women in relation to the total number of urine culture episodes per woman. %: the percentage of women in relation to the 8,807 women that had one or more urine culture episodes.

Most women examined had one, two, or three urine cultures analyzed during pregnancy, 52% (4,589/8,807), 25% (2,181/8,807), and 12% (1,040/8,807), respectively. The maximum number of urine cultures analyzed in one woman was 21 (Figure 2).

**Figure 3:**
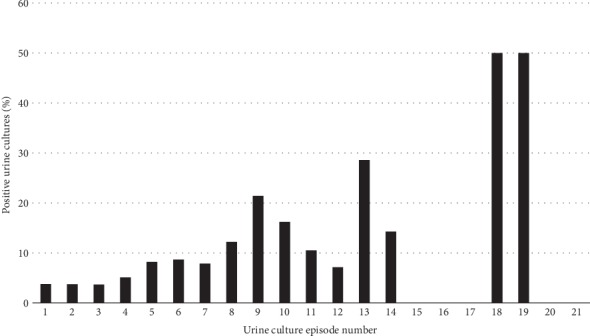
The proportion of positive urine cultures in relation to the urine culture episode number in pregnant women.

4.3% of all urine cultures were positive with a modest increase in the incidences of positive culture findings in relation to sampling rate, especially with multiple urine cultures performed (Figure 3).

The most frequent bacteria isolated in the urine cultures are shown in Table 1. The table shows the causative pathogens found in all the urine samples, including the repeated samples in the cohort of pregnant women.

**Figure 4:**
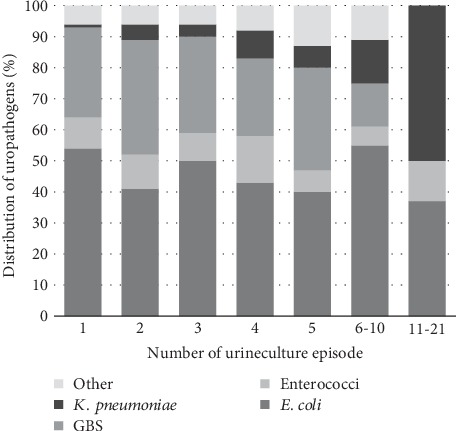
The distribution of the most common uropathogen found in repeated urine cultures. Others include all other significant uropathogen found aside the most frequent ones outlined in the figure.

*Klebsiella pneumoniae* was found as a dominant uropathogen in patients that had multiple urine cultures analyzed (Figure 4).

Using the study criteria for a positive urine culture, a total of 734 positive urine cultures originating from 497 women were identified (Figure 1). The overall incidence of bacteriuria defined as one or more episodes of bacteriuria in pregnancy was 5.6% in the group of women with urine culture tests.

Of the 497 women with bacteriuria, 78% (386/497) had only one episode during pregnancy, 12% (62/497) had two, and 4% (21/497) had three episodes, with the remaining six percent (28/497) of women in the cohort having four or more bacteriuria episodes during pregnancy.

Women below the age of 25 had an increased likelihood of bacteriuria OR_a_ 1.60 (CI 1.26 to 2.02, *p* < 0.001) compared with women aged 25-34, similar was found in women age > 34, OR_a_ 1.28 (CI 1.01 to 1.61, *p* = 0.040). In reference to Caucasians, certain ethnic groups were found to have an increased likelihood of bacteriuria; Afro Caribbean origin OR_a_ 1.872 (CI 1.13 to 3.07, *p* = 0.014), Asian origin OR_a_ 2.07 (CI 1.29 to 3.32, *p* = 0.002), and mixed ethnicity OR_a_ 2.34 (CI 1.23 to 4.46, *p* = 0.010). Women delivering preterm were more likely to have had an episode of bacteriuria during pregnancy than women delivering week 35 or later OR 2.05 (CI 1.36 to 3.09 *p* = 0.001).

The selected predictors of bacteriuria are shown in Table 2.

## 4. Discussion

This study is, to our knowledge, the first to describe the frequency of urine cultures performed in relation to bacteriuria during pregnancy.

The key finding in this study is that 42% of the general pregnant population in Aarhus has a urine culture performed at least once during pregnancy and nearly half of them more than one time. A total of 96% of all urine cultures were negative. Initiatives for optimized collection of urine samples should be supported. Such initiatives could be correct hygienic procedures during urine sample collection. This would reduce the numbers of leucocyte positive dipsticks and thus the number of samples sent for culturing. A reduction in time used in the laboratory and money for culturing could be expected.

We found an incidence of 5.6% of bacteriuria in the cohort, which is high in comparison to previous reports in Danish studies. A retrospective study performed more than 40 years ago, including 15,626 women, found a bacteriuria incidence of 2.8% in gestational weeks 28–30 [17]. Another study, including 4,274 women, found an incidence of bacteriuria of 2.9% in gestational weeks 14–18 [18]. Both studies excluded all women with symptoms and their results reflected only asymptomatic bacteria and not bacteriuria in general. The explanation for the higher incidence of bacteriuria in our study may be that some of the urine samples are sent for analysis as a result of a positive dipstick or positive microscopy. The sample are sent to the Department of Clinical Microbiology from the outpatient pregnancy clinic, the labor ward, or from the GP. Unfortunately, information regarding the indication for the analysis is not available. Thus, it is not merely asymptomatic bacteriuria but also symptomatic bacteriuria being tested.

When analyzing the data, we identified subgroups of women with an increased likelihood of bacteriuria during pregnancy. These groups were pregnant women <25 and> 34 years of age and women with Afro-Caribbean or Asian origin and mixed ethnicity. Knowledge of this might lead to more attention to these women when diagnosing bacteriuria in pregnancy.

Women who give birth in gestational weeks 28–34 were also more likely to have bacteriuria during pregnancy. This finding is consistent with urinary tract infections being associated with preterm delivery. According to the instruction in our department and in Denmark in general, urine cultures are always sent for culture in pregnant women with threatening preterm delivery. One could argue that this makes it more likely to diagnose bacteriuria in this subgroup of women.

Our uropathogen findings differ somewhat from findings previously reported in international literature. As others, we find that *E*. *coli* is the predominant uropathogen in bacteriuria. But where others have reported *E*. *coli* in up to 80% of cases [1, 2, 13], we only isolated *E*. *coli* in 49% of all positive urine cultures. A former Danish study reports *E*. *coli* isolated in 57% of the women with bacteriuria, which correlates to our findings [18]. In contrast with this, GBS was more frequently isolated in the urine of pregnant women in our study than reported internationally [1, 11]. This difference could be explained in part by the lower thresholds for reporting GBS in cultures.

The Danish antenatal guideline recommends treating bacteriuria in pregnancy regardless of the symptoms, and with the antibiotic, pivmecillinam, as the preferred choice of treatment. The recommended choice of antibiotic treatment of GBS bacteriuria is penicillin [19]. If GBS bacteriuria is diagnosed in pregnancy, intrapartum antibiotic prophylaxis is recommended because of the risk of neonatal GBS infection [15].

The main strength of our study is the large number of pregnant women included over a long period of time, combined with the solid foundation of national databases and social security numbers established in Denmark, which provide the comprehensive exploration of retrospective data.

Our findings, however, are challenged regarding transferability to the general obstetric population, because our data only represents those with urine cultures at the microbiological department. These urine samples originate from patients in either a hospital setting or at the general practitioner as part of the antenatal care based on symptomatology and/or microscopy and urine dipstick. It is well known that some general practitioners perform their own urine cultures locally, without the assistance from the microbiological department. Consequently, there are an uncertain number of bacteriuria episodes in the cohort of pregnant women that are not included in our data.

Even in Denmark, a small country with 6 million inhabitants, the procedures around urine sampling and analysis and treatment on suspicion of urinary tract infection varies. This variation is most likely to be greater in an international perspective.

We hope that the information from our study will increase the focus in Denmark and hopefully in other comparable countries on urine sampling technique to reduce the number of samples sent for culture and the number of pregnant women unnecessarily treated on suspicion of urinary tract infections.

Information regarding additional risk factors such as low socioeconomic status, diabetes, history of urinary tract infections, and urinary tract abnormalities in the cohort would have been very valuable. This information would be of great interest for further research.

Furthermore, it would be interesting to find information on all antibiotic prescriptions targeting uropathogens in an attempt to reevaluate the effect of bacteriuria in the pregnant Danish women cohort. The challenge for this would be the uncertainty with regard to medicine compliance.

In conclusion, a total of 42% of women had a urine culture performed at least once during pregnancy; only 4% of all urine cultures were positive. The most frequent bacteria isolated in the urine cultures was *E*. *coli,* which were isolated in 49%, followed by *S*. *agalactiae* (GBS) 29% and *Enterococci* 10%. The overall incidence of bacteriuria defined as one or more episodes of bacteriuria in pregnancy was 5.6% in the group of women with urine culture samples. Women of a younger age, women from certain ethnical groups, and women delivering preterm appear to be more likely to have bacteriuria during pregnancy, suggesting that increased attention to these groups would be beneficial when diagnosing bacteriuria during pregnancy.

## Figures and Tables

**Table 1:** Causative agents of bacteriuria among the 497 pregnant women with one or more episodes of bacteriuria.

Uropathogen (species)	No (%)
*Escherichia coli*	372 (49)
*Streptococcus agalactiae* (GBS)	223 (29)
*Enterococcus species*	75 (10)
*Klebsiella pneumoniae*	38 (5)
*Klebsiella oxytoca*	4 (0.5)
*Enterobacter cloacae*	8 (1)
*Enterobacter aerogenes*	7 (1)
*Enterobacter species*	1 (0.1)
*Staphylococcus saprophyticus*	9 (1)
*Staphylococcus aureus*	7 (1)
*Citrobacter koseri*	6 (1)
*Proteus mirabilis*	4 (0.5)
*Proteus vulgaris*	1(0.1)
*Pseudomonas aeruginosa*	3 (0.4)
*Serratia marcescens*	1(0.1)
Total	759 (100)

Bacteriuria definition: 1 × 10^5^ CFU per ml of a single or two microorganisms. For GBS, 1 × 10^4^.

**Table 2:** Predictors of bacteriuria in the cohort of pregnant women.

Predictors	Bacteriuria *N* = 497 (%)	No bacteriuria *N* = 20,437	Total *N* = 20,934	Odds ratio (OR_c_ and OR_a_)	Confidence interval 95%	*p* value
Maternal age (years)						
<25	92 (3.37)	2,636	2,728	1.62	1.28-2.05	0.000
				*1.60*	*1.26–2.02*	*0.000*
25-34 (reference)	310 (2.11)	14,384	14,694	1		
>34	95 (2.71)	3,417	3,512	1.29	1.02-1.63	0.032
				*1.28*	*1.01–1.61*	*0.040*
Currently smoking						
No (reference)	455 (2.33)	19,049	19,504	1		
Yes	42 (3.21)	1,268	1,310	0.93	0.78-1.09	0.356
Unknown	0	120	120			
BMI						
<19	307 (2.31)	12,967	13,274	0.98	0.69-1.40	0.920
20-24 (reference)	35 (2.26)	1,511	1,546	1		
25-29	92 (2.41)	3,722	3,814	1.05	0.83-1.33	0.696
>29	56 (2.88)	1,891	1,947	1.26	0.94-1.68	0.122
Unknown	7 (1.98)	346	353			
Ethnicity						
Caucasian (reference)	433 (2.25)	18,826	19,259	1		
Asian	19 (4.57)	397	416	2.08	1.30-3.33	0.002
				*2.07*	*1.29–3.32*	*0.002*
Oriental	12 (3.64)	318	330	1.64	0.91-2.94	0.097
				*1.67*	*0.93–2.99*	*0.087*
Afro-Caribbean	17 (4.39)	370	387	2.00	1.22-3.28	0.006
				*1.87*	*1.13–3.07*	*0.014*
Mixed ethnicity	10 (5.26)	180	190	2.42	1.27-4.60	0.007
				*2.34*	*1.23–4.46*	*0.010*
Unknown	6 (1.70)	346	352	0.75	0.33-1.70	0.496
				*0.72*	*0.32–1.62*	*0.422*
Parity						
Nullipara (reference)	255 (2.32)	10,735	10,990	1		
Multipara	202 (2.48)	7,945	8,147	1.07	0.89-1.29	0.476
Unknown	40 (2.23)	1,757	1,797	0.96	0.68-1.34	0.805

Results from the multivariable analysis are set in italic. %: the percentage of women with bacteriuria in relation to the total number of women with the given predictor. Asian: south Asian, descendants from countries around Pakistan, Bangladesh, and India. Oriental: descendants from China, Japan, and Korea. Mixed: different ethnicity of the mother and father of the pregnant woman.

## Data Availability

The register data used to support the findings of this study are restricted by the Danish patient safety authority in order to protect personal data. Anonymized data are available from the corresponding author upon request.

## Abbreviations

BMI: Body mass index
CFU: Colony-forming units
DFMD: Danish fetal medicine database
GBS: Group B streptococci
MADS: Microbiological database at the Department of Clinical Microbiology at Aarhus University Hospital
OR_c_: Crude odds ratio
OR_a_: Adjusted odds ratio.
